# Blood volume and hemoglobin mass in long-term heart transplant recipients with and without Anemia

**DOI:** 10.1186/s13019-021-01510-1

**Published:** 2021-06-02

**Authors:** Florian Seiler, Christoph Ahlgrim, Philipp Birkner, Nina Wrobel, Jonathan Rilinger, Sebastian Grundmann, Christoph Bode, Torben Pottgiesser

**Affiliations:** 1 Department of Cardiology and Angiology I, Heart Center, Faculty of Medicine, University Medical Center Freiburg, University of Freiburg, Hugstetter Str. 55, 79106 Freiburg, Germany; 2 Department of Cardiology and Angiology II, Heart Center, Faculty of Medicine, University Medical Center Freiburg, University of Freiburg, Bad Krozingen, Germany; 3 Center for Medicine, Institute for Exercise- and Occupational Medicine, Medical Center, Faculty of Medicine, University of Freiburg, Freiburg, Germany

**Keywords:** Heart transplantation, Anemia, Hemoglobin mass, Red cell volume, Plasma volume, Blood volume

## Abstract

**Background:**

In systolic chronic heart failure, a heterogeneous blood volume (BV) regulation can be found with plasma volume expansion in many cases, possibly leading to pseudoanemia. Little is known about the volume status after heart transplantation (HTX). So far, anemia of HTX recipients was solely investigated using hemoglobin-concentration that may be misleading in a clinical context. The objective of the study was whether a difference in plasma volume and red cell volume can be observed in clinically stable heart transplant recipients compared to matched control subjects. Secondary, the aim was to describe anemia in the long-term after HTX based on quantitative data.

**Methods:**

Blood volume and its constituents red cell volume and plasma volume were quantified using an abbreviated carbon monoxide rebreathing method (aCORM) with focus on its primary measure total hemoglobin mass (Hbmass) and coincidental anemia in 36 (7 women) heart transplant recipients. For comparison, a matched control group of 46 (5 women) healthy subjects was selected.

**Results:**

Neither Hbmass nor blood volumes were significantly different in HTX patients compared to matched healthy control group subjects. The prevalence of anemia 6.3 ± 4.3 years after transplantation was 19%. Hbmass and red cell volume were significantly lower in anemic HTX patients compared to non-anemic patients while plasma volume was not expanded. Various immunosuppressant regimens did not have an effect on Hbmass, plasma volume or red cell volume.

**Conclusions:**

There was no difference in blood volumes and Hbmass between HTX patients and control subjects. The pathophysiologic blood volume regulation in chronic heart failure does not seem to be longer active in long-term HTX recipients. However, in the long-term after HTX, anemia occurs in a considerable number of patients as true anemia without a clear association with immunosuppression.

**Trial registration:**

German registry for clinical studies, DRKS00006078. Registered 09 May 2014, https://www.drks.de/drks_web/navigate.do?navigationId=trial. HTML&TRIAL_ID=DRKS00006078.

## Background

In chronic heart failure (CHF), the composition of blood volume (BV) continues to be of interest to fully understand pathophysiological adaptations [1, 2]. Quantitative measurement of total BV was considered essential to identify the heterogeneity of BV regulation [1]. After heart transplantation (HTX), that can be considered the definitive treatment for CHF, there is limited data available concerning the quantitative assessment of BV. In small studies, extracellular fluid volume expansion was found in clinically stable heart transplant recipients who became hypertensive [3, 4]. This was explained to some extent by a failure to suppress the renin-angiotensin-aldosterone system (RAAS) when hypervolemia occurs after heart transplantation [5].

Volume overload in CHF is typically described as plasma volume (PV) expansion, conceivably leading to hemodilution and pseudoanemia [6–8]. It is of particular importance that not all patterns of volume overload are the same, and a marked heterogeneity between CHF patients can be observed [1, 9]. True anemia in the sense of a reduced red cell volume (RCV) below normal is also possible [10]. Reflecting on the complex pathophysiology, our group recently described an increased RCV as a “relevant contributing factor to hypervolemia in addition to an expanded PV in compensated chronic heart failure with reduced ejection fraction (EF)” [11].

On the other hand, anemia after heart transplantation was suggested to have prognostic value [12, 13] but its prevalence seems to vary in studies using different anemia definitions [13–15] and time points of evaluation. These data were relying on hemoglobin concentration [Hb], which may be misleading in a clinical context. There is no quantitative data available regarding anemia after HTX.

Clinicians frequently use radioactively labeled albumin for assessment of intravascular volumes [9, 10]. In the context of heart transplantation, PV was determined by using a modified Evans blue dye (T-1824) dilution technique [5] and radioiodine-labeled serum albumin [4]. In addition, rebreathing of carbon monoxide (CO) to determine hemoglobin mass (Hbmass) and RCV and PV can be applied using different protocols [16, 17]. The abbreviated protocol (aCORM) [17] was used in applied physiology and increasingly in a clinical environment [18].

The objective of the study was whether a difference in PV and RCV can be observed in clinically stable long-term heart transplant recipients compared to matched control subjects applying aCORM. Secondary, the aim was to describe coincidental anemia in the long-term after HTX based on quantitative BV data.

## Methods

### Study design

In a cross-sectional, controlled approach, Hbmass, RCV and PV were measured in 39 patients (8 women) after heart transplantation. The patients (Group HTX) were recruited upon their routine follow-up visit in the transplantation unit of our heart center after providing signed informed consent. Inclusion criteria were a period of at least 6 months since transplantation and clinical stability without any sign for organ rejection. Exclusion criteria were hemodynamic instability, acute coronary syndrome, anemia with a hemoglobin concentration ([Hb]) ≤ 8 g/dl, active bleeding, active malignancy, limited life expectancy < 1 year, noncardiac chronic renal disease, uncontrolled lung disease, acute infection and chronic inflammatory disease. Associated data such as echocardiography and laboratory results, medication and functional status were recorded in the context of the visit. The subjects were deemed “stable” based on their routine follow up visit free of cardiac symptoms and without any sign for organ rejection.

A healthy control group (Group CON) with no obvious medical condition and no established medication was matched retrospectively by age, body weight and height from our database of reference subjects yielding 46 subjects (5 women) whose Hbmass and BV were previously measured using the aCORM in our laboratory. This group was selected only for the comparison of Hbmass and BV data. The same exclusion criteria applied to the control group.

The study was approved by the ethics committee of our University Hospital (31/14) and is in line with the latest revised form of the Declaration of Helsinki. The study is registered in the German registry for clinical studies (DRKS-ID: DRKS00006078).

### Determination of laboratory results

Venous blood from HTX patients was analyzed for [Hb] and Hct, proBNP, iron, ferritin, and transferrin saturation as well as plasma levels of immunosuppressive agents among other values. Vitamin B12 was not ordered on a routine basis. In CON, venous [Hb] and Hct were available in the database from the time of application of aCORM.

### Determination of hemoglobin mass and blood volumes

Hbmass and subsequently, blood volumes were derived applying aCORM [17]. As CO has a strong affinity for hemoglobin (Hb), it can be used to label Hb molecules by inhalation as CO holds excellent lung diffusion capacities. The dilution of labeled Hb molecules in unlabeled Hb molecules enables calculation of the total number of Hb molecules and thus their dimension Hbmass as the primary measured quantity according to the formula mentioned below following to the indicator-dilution principle. Subjects were in a seated position for at least ten minutes before commencing the rebreathing maneuver. The essential steps of the test procedure were practiced before execution. A defined CO bolus (0.7–0.8 ml/kg body weight) is deeply inhaled with a pause for breath for 10 s and rebreathed for 110 s after mixture with oxygen using a closed-circuit spirometer (SpiCO, Blood Tec, Germany). A portable gas analyzer (Draeger Pac 7000, Draeger, Germany) was used to detect CO leakages at the mouthpiece and the spirometer during the procedure. The spirometer then was disconnected. The amount of CO remaining in the rebreathing circuit and spirometer was quantified. Carboxyhemoglobin concentration [COHb] was measured in capillary blood before as well as six and eight minutes after administration of CO. Capillary sampling was performed from the earlobe using a hyperemizing ointment (Finalgon 4 mg/g + 25 mg/g, Nonivamid and Nicoboxil, Boehringer Ingelheim, Germany) to standardize blood-sampling conditions. Each sample was drawn into a capillary tube with a volume of 55 μl (Radiometer Clinitubes 55 μl, Radiometer, Denmark) and immediately analyzed using a point-of-care blood gas analyzer (in HTX Radiometer ABL 700series, Radiometer, Denmark, in CON Radiometer OSM3, Radiometer, Denmark) for determination of [COHb] and capillary [Hb]. Two additional capillary samples were drawn into two further capillary tubes, each with a volume of maximal 50 μl (Hettich Standard, Hettich, Germany) and immediately used for determination of Hct using a centrifuge (Hettich Mikro 20, Hettich, Germany). Hbmass was calculated from the mean change in [COHb] as described previously [17] and outlined here:

$$ \mathrm{Hbmass}=\mathrm{K}\times {\mathrm{M}}_{\mathrm{CO}}\times 100\times {\left(\Delta \mathrm{HbCO}\%\times 1.39\right)}^{-1} $$

where.

K = current barometric pressure × 760^− 1^ × [1 + (0.003661 × current temperature)].

M_CO_ = CO_administered_ (CO_system + lung (after disconnection)_ + CO_exhaled (after disconnection)_).

CO_administered_ = CO volume administered into the spirometer.

CO_system + lung (after disconnection)_ = CO concentration in the spirometer × (spirometer volume + lung residual volume).

CO_exhaled (after disconnection)_ = end-tidal CO concentration × alveolar ventilation × time.

ΔHbCO% = difference between baseline HbCO and HbCO in the blood samples after CO administration.

1.39  Hüfner’s number (ml CO × Hb^− 1^)

End tidal CO concentration and CO concentration in the spirometer were directly determined using a portable CO analyzer (Draeger Pac 7000, Draeger, Germany). Alveolar ventilation and lung residual volume were approximated based on the subject’s characteristics.

Alveolar ventilation and lung residual volume were approximated based on the subject’s characteristics.

Units: Hb: g; current barometric pressure: mmHg; current temperature: °Celsius; CO: ml; CO concentration: ppm.

Intravascular volumes (RCV, PV, BV) can be then calculated using the following formulas [19]:

$$ \mathrm{RCV}=\mathrm{Hbmass}/\mathrm{MCHC}\ast 100 $$

$$ \mathrm{BV}=\mathrm{RCV}\ast 100/{\mathrm{Hct}}^1 $$

$$ \mathrm{PV}=\mathrm{BV}-\mathrm{RCV} $$

MCHC = mean corpuscular hemoglobin concentration, venous [Hb] and Hct were used for determination of MCHC. For RCV calculation, Hct was corrected to whole-body Hct by the factor 0.91 [20] (Hct^1^). As venous [Hb] was unavailable in four HTX patients, it was inferred from capillary [Hb] by a factor based on regression analysis of venous and capillary [Hb] in the remaining HTX subjects. Based on the recommendation of the International Council for Standardization in Hematology (ICSH), intravascular volumes and Hbmass were adjusted to body surface area (BSA) [21] to present data for better comparison. BSA (m^2^) was estimated by using the Du Bois formula [22].

For both groups, the expected normal blood volumes were calculated according to the gender-specific ICSH formulae [21] to analyze subjects in both groups regarding their predicted BV.

Anemia was defined according to the definition of the World Health Organization (WHO) with [Hb] < 13 g/dl in men and [Hb] < 12 g/dl in women [23]. GFR was estimated using the equation suggested by the Modification of Diet in Renal Disease Study Group (MDRD) [24].

EF was determined through the modified biplane Simpson’s rule [25] if possible based on image quality. In case of insufficient image quality, EF was estimated by thorough visual analysis of regional wall motion and classification from the standard long axis parasternal view, short axis parasternal view as well as apical four and two chamber view.

### Statistical analysis

SAS JMP 9.0 and Graphpad Prism 6.0f were used for data administration and statistical analyses. A two-tailed t-test was used to test for group differences. A two-tailed chi-square test was used for the analysis of contingency tables regarding anemia. An ordinary one-way ANOVA was used to test the effect of the immunosuppressant regimen on Hbmass and blood volumes. The prevalence of anemia was analyzed using Fischer’s exact test. The alpha level was set at 0.05.

## Results

In HTX, three patients (one woman) had to be excluded due to insufficient test performance (e.g. lack of understanding of the instructions) and leakages at the mouthpiece. In the final analysis, 36 (7 female) HTX patients and 46 (5 female) CON subjects were included. All subjects tolerated aCORM well. There were no adverse cardiac effects, such as chest pain, dizziness or shortness of breath, and no signs of CO toxicity such as headaches or visual disturbances. The [COHb] before CO rebreathing was 1.1 ± 0.7% in HTX and 1.0 ± 0.9% in CON. At the time “6 min” after the commencement of CO rebreathing [COHb] increased to 6.3 ± 1.3% in HTX and 6.8 ± 1.2% in CON.

The baseline characteristics of both groups including gender-specific data are displayed in Table 1. There were no significant differences between both groups regarding anthropometric data or distribution of age. In HTX, time since transplantation was 6.3 ± 4.3 years with a normal function of the transplanted heart assessed by EF of 59.7 ± 6.7%. Medical therapy and the immunosuppressant regimen of HTX are displayed in Table 1. The large standard deviation of proBNP is based on three outliers with values above 1500 pg/ml, who were clinically asymptomatic.

**Table 1:** Baseline characteristics of heart transplant recipients and healthy control subjects

	Group		p
	HTX	CON	
Sex (F, M, total n)	7 F, 29 M, 36	5 F, 41 M, 46	
Age (yrs)	51.6 ± 15.3	52.1 ± 13.4	0.878
Height (cm)	177.3 ± 8.0	178.4 ± 6.4	0.531
Weight (kg)	83.9 ± 18.1	83.0 ± 10.7	0.773
BMI (kg/m2)	26.6 ± 5.0	26.1 ± 3.6	0.676
BSA (m2)	2.0 ± 0.2	2.0 ± 0.1	0.944
Heart rate (/min)	84.7 ± 10.9	–	
Mean arterial pressure (mmHg)	104 ± 10.7 (*n* = 32)	–	
Ejection fraction (%), Simpson’s rule	59.7 ± 6.7 (*n* = 29)	–	
E/e’ mean	9.2 ± 4.1 (*n* = 31)		
proBNP (pg/ml)	555 ± 525 (n = 32)	–	
Years since heart transplantation	6.3 ± 4.3	–	
**Cause for HTX**			
Ischemic CM, n (%)	9 (25)	–	
Dilatative CM, n (%)	20 (56)	–	
Myocarditis, n (%)	5 (14)	–	
Other, n (%)	2 (5)	–	
**Medication in HTX**			
Calciumchannel blocker	19 (53)		
Beta-blocker	8 (22)	–	
Ivabradine	1 (3)	–	
ACE-inhibitor	15 (42)	–	
AT-1 antagonist	9 (25)	–	
Diuretic	19 (53)	–	
Loop diuretic	16 (44)	–	
Thiazide	4 (11)	–	
Iron supplementation	3 (8)	–	
Glucocorticoid	3 (8)	–	
**Immunosuppression**			
Everolimus / Cyclosporine	18 (50)	–	
Everolimus / Tacrolimus	7 (19)	–	
MMF / Cyclosporine	7 (19)	–	
MMF / Tacrolimus	4 (11)	–	

**Footnote**: Data presented as mean ± SD. HTX = heart transplant recipients. CON = control subjects. BMI = body mass index. BSA = body surface area. proBNP = pro – brain natriuretic peptide (normal range < 125 pg/ml). CM = cardiomyopathy. ACE = angiotensin converting enzyme. AT-1 = angiotensin 1. MMF = mycophenolate mofetil

Hbmass as well as RCV were not different between HTX and CON, neither in the absolute nor BSA-adjusted analysis (Table 2). PV was not significantly different in both groups albeit in HTX there was a trend towards an increased PV which was significant in the small subset of women. Accordingly, venous [Hb] and Hct were significantly lower in HTX compared to CON. The total BV was similar in both groups (Table 2). The calculated normal values for BV parameters did not vary between HTX and CON.

**Table 2:** Blood volume data and selected laboratory data of heart transplant recipients and healthy control subjects

	All subjects					Men					Women				
	HTX	N	CON	N	p	HTX	N	CON	N	p	HTX	N	CON	N	p
**Absolute**															
Hbmass (g)	785 ± 221	36	806 ± 121	46	0.597	843 ± 195	29	832 ± 100	41	0.781	543 ± 149	7	597 ± 59	5	0.409
RCV (ml)	2341 ± 653	36	2351 ± 345	46	0.938	2500 ± 572	29	2425 ± 280	41	0.517	1684 ± 579	7	1743 ± 189	5	0.809
PV (ml)	3871 ± 846	36	3649 ± 513	46	0.171	4012 ± 703	29	3757 ± 430	41	0.090	3289 ± 1175	7	2767 ± 156	5	0.289
BV (ml)	6212 ± 1433	36	6000 ± 801	46	0.428	6512 ± 1198	29	6182 ± 636	41	0.183	4973 ± 1750	7	4510 ± 230	5	0.514
**BSA-corrected**															
Hb mass (g/m2)	386 ± 85	36	400 ± 45	46	0.387	407 ± 80	29	410 ± 35	41	0.873	299 ± 25	7	317 ± 23	5	0.222
RCV (ml/m2)	1152 ± 243	36	1165 ± 125	46	0.758	1208 ± 231	29	1195 ± 94	41	0.776	919 ± 132	7	924 ± 72	5	0.932
PV (ml/m2)	1919 ± 208	36	1814 ± 222	46	0.089	1948 ± 304	29	1855 ± 195	41	0.155	1798 ± 317	7	1472 ± 114	5	**0.037**
BV (ml/m2)	3071 ± 505	36	2979 ± 312	46	0.344	3156 ± 488	29	3050 ± 246	41	0.289	2717 ± 443	7	2396 ± 114	5	0.110
**Calculated normal (ICSH)**															
RCV (ml)	2097 ± 369	36	2123 ± 250	46	0.713	2232 ± 232	29	2186 ± 181	41	0.376	1537 ± 301	7	1609 ± 81	5	0.563
PV (ml)	3104 ± 422	36	3135 ± 258	46	0.696	3246 ± 246	29	3197 ± 192	41	0.376	2515 ± 504	7	2627 ± 128	5	0.560
BV (ml)	5201 ± 788	36	5258 ± 508	46	0.703	5478 ± 478	29	5383 ± 373	41	0.376	4052 ± 805	7	4236 ± 209	5	0.560
**Additional data**															
[Hb] (g/dl)	13.8 ± 1.6	36	14.8 ± 0.9	46	**0.002**	14.1 ± 1.5	29	14.8 ± 0.9	41	**0.043**	12.2 ± 1.2	7	14.5 ± 1.1	5	**0.006**
Hct (%)	41.0 ± 4.0	36	43.1 ± 2.7	46	**0.012**	41.9 ± 3.9	29	43.1 ± 2.7	41	0.161	37.3 ± 1.6	7	42.4 ± 3.2	5	**0.020**
Iron (μg/dl)	74 ± 28	33	**–**		**–**	75 ± 25	27	**–**		**–**	69 ± 40	6	**–**		**–**
Ferritin (ng/ml)	138 ± 139	31	**–**		**–**	144 ± 146	25	**–**		**–**	113 ± 110	6	**–**		**–**
Transferrin saturation (%)	20 ± 7	33	**–**		**–**	21 ± 7	27	**–**		**–**	18 ± 7	6	**–**		**–**

Footnote: Data presented mean ± SD. HTX = heart transplant recipients. CON = control subjects. BSA = body surface area. Hbmass = total hemoglobin mass. RCV = red cell volume. PV = plasma volume. BV = blood volume. *calculated normal values according to ICSH formulae (sex-dependent). [Hb] = venous hemoglobin concentration. Hct = venous hematocrit. N indicates the number of patients/subjects for whom data was available. p indicates the *p*-value (bold numbers indicate statistical significance)

**Fig. 1:**
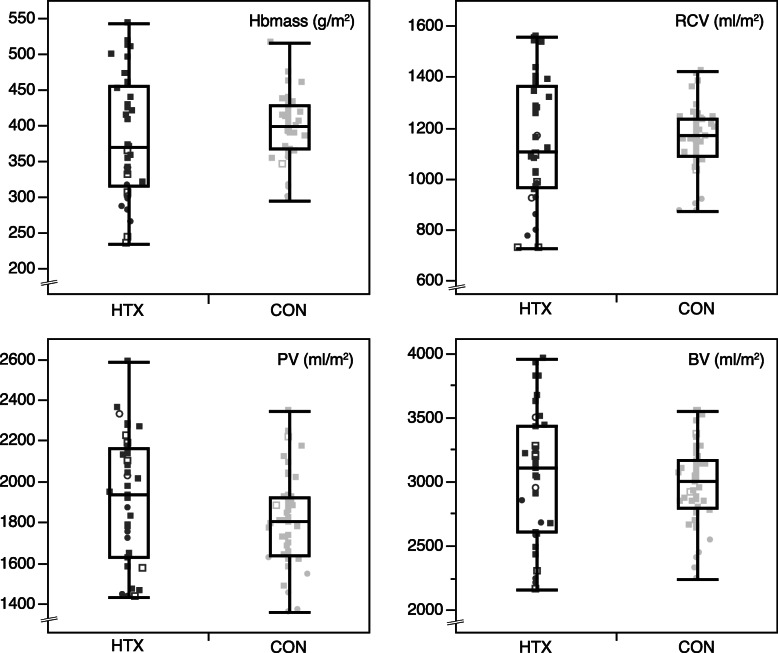
Comparison of heart transplant recipients (HTX) and healthy control subjects (CON) with individual data (grey shades, women are displayed as circles, men as squares. Anemia according to WHO definition is expressed by empty symbols) and boxplots of body-surface corrected hemoglobin mass (Hbmass), red cell volume (RCV), plasma volume (PV) and blood volume (BV)

Figure 1 shows individual data of BSA-adjusted Hbmass and blood volumes. In HTX, diuretics were used in 53% of patients (Table 2). In a subgroup analysis, there was no difference of HTX patients with diuretics (44%) against patients without diuretics (56%): Hbmass (*p* = 0.997), RCV (*p* = 0.989), PV (*p* = 0.370) and BV (*p* = 0.594). When analyzing HTX patients with (67%) or without (33%) RAAS-inhibition, PV was significantly lower with RAAS-inhibition (*P* = 0.037) while Hbmass, RCV and BV were indifferent.

**Fig. 2:**
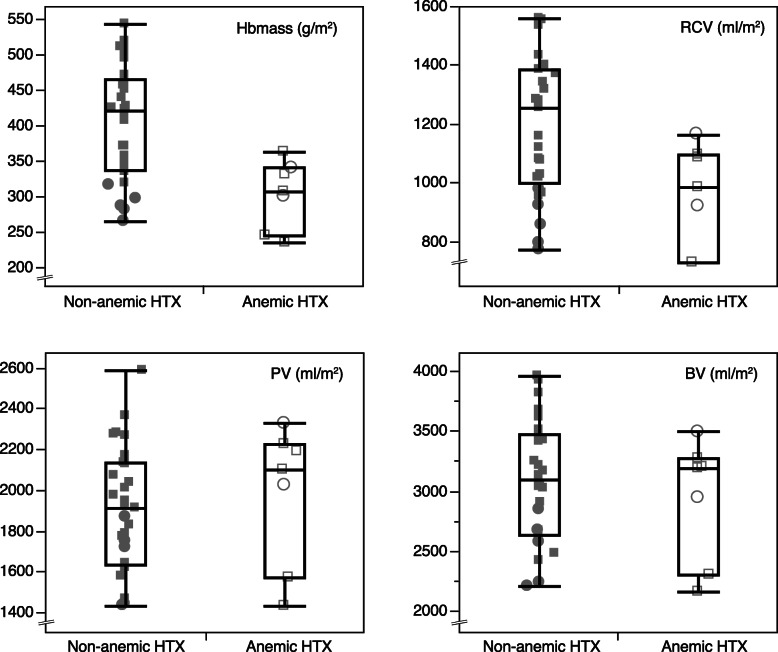
Non-anemic and anemic heart transplant recipients (HTX) with individual data (grey shades, women are displayed as circles, men as squares. Anemia according to the WHO definition is expressed by empty symbols) and boxplots of body-surface corrected hemoglobin mass (Hbmass), red cell volume (RCV), plasma volume (PV) and blood volume (BV)

When defined by the WHO criteria, anemia was more common in HTX (7 patients (19%)) than in CON (2 subjects (4%), *p* = 0.038). The details of the anemic HTX patients are presented in Table 3 and Fig. 2. Anemic HTX patients yielded significantly reduced Hbmass and RCV (BSA-corrected values). PV and BV were not significantly different between anemic and non-anemic HTX patients. This reduction of Hbmass and RCV translated into significantly reduced venous [Hb] and Hct. Anemic HTX patients had significantly lower iron and transferrin saturation while vitamin D, ferritin and CRP were indifferent compared to non-anemic patients.

**Table 3:** Blood volume data and selected laboratory data of heart transplant recipients with and without anemia

	HTX				
	Anemic	N	Non-anemic	N	p
**Absolute**					
Hbmass (g)	593 ± 168	7	831 ± 209	29	**0.009**
RCV (ml)	1892 ± 615	7	2450 ± 624	29	0.059
PV (ml)	3893 ± 1195	7	3866 ± 767	29	0.955
BV (ml)	5785 ± 1802	7	6316 ± 1347	29	0.487
**BSA-corrected**					
Hb mass (g/m2)	303 ± 48	7	406 ± 80	29	**< 0.001**
RCV (ml/m2)	961 ± 176	7	1198 ± 236	29	**0.012**
PV (ml/m2)	1984 ± 343	7	1903 ± 303	29	0.580
BV (ml/m2)	2945 ± 511	7	3101 ± 508	29	0.488
**Additional data**					
[Hb] venous (g/dl)	11.4 ± 0.8	7	14.3 ± 1.2	25	**< 0.001**
Hct venous (%)	35.8 ± 1.5	7	42.8 ± 3.3	25	**< 0.001**
Erythrocytes (Mio/μl)	4.5 ± 0.5	7	5.2 ± 0.5	25	**0.007**
MCV	80.0 ± 6.1	7	82.1 ± 4.9	25	0.431
MCH	25.4 ± 2.8	7	27.5 ± 1.8	25	0.108
MCHC	31.7 ± 1.6	7	33.5 ± 0.8	25	**0.024**
Iron (μg/dl)	48 ± 18	7	81 ± 26	26	**0.002**
Ferritin (ng/ml)	198 ± 269	7	123 ± 88	25	0.531
Transferrin saturation (%)	15 ± 6	6	21 ± 7	26	**0.044**
Vitamin D (ng/ml)	24 ± 15	6	25 ± 14	24	0.902
CRP (mg/l)	6.6 ± 4.2	5	9.9 ± 7.4	14	0.246
Serum-creatinine (mg/dl)	1.4 ± 0.4	7	1.2 ± 0.4	25	0.335
GFR (ml/min)	59 ± 29	7	73 ± 28	25	0.306
Urea (mg/dl)	62 ± 28	7	45 ± 26	25	0.182

Footnote: Data presented mean ± SD. HTX = heart transplant recipients. BSA = body surface area. Hbmass = total hemoglobin mass. RCV = red cell volume. PV = plasma volume. BV = blood volume. [Hb] = venous hemoglobin concentration. Hct = venous hematocrit. MCV = mean corpuscular volume. MCH = mean corpuscular hemoglobin. MCHC = mean corpuscular hemoglobin concentration. CRP = C-reactive protein. GFR = Glomerular filtration rate. N indicates the number of patients/subjects for whom data was available. p indicates the p-value (bold numbers indicate statistical significance)

**Fig. 3:**
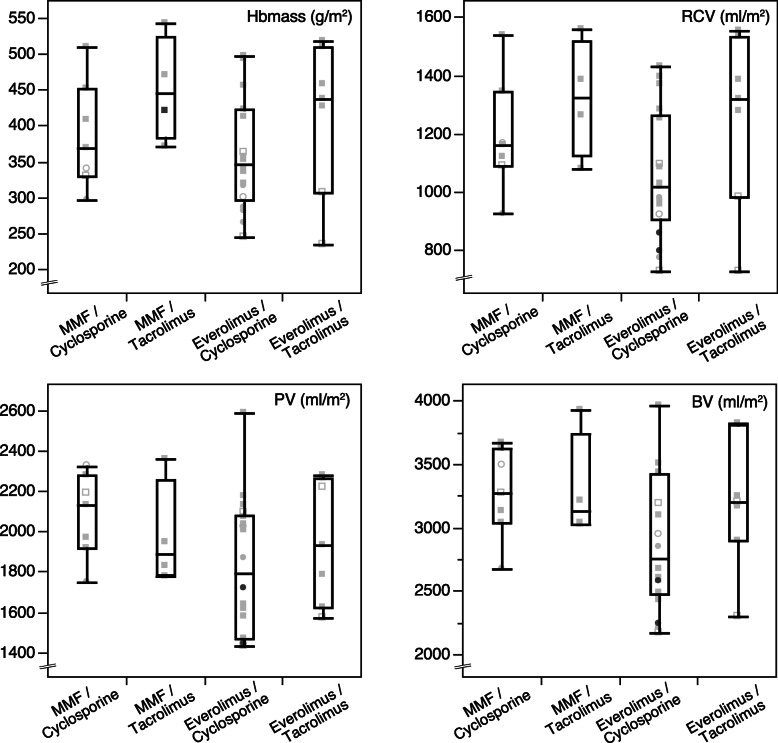
Effect of immunosuppressant regimens with individual data (grey shades, women are displayed as circles, men as squares. Anemia according to WHO definition is expressed by empty symbols) and boxplots of body-surface corrected hemoglobin mass (Hbmass), red cell volume (RCV), plasma volume (PV) and blood volume (BV). MMF = mycophenolate mofetil

There was no significant effect of the type of immunosuppressant regimen on Hbmass (*p* = 0.139), RCV (*p* = 0.182), PV (*p* = 0.249) and BV (*p* = 0.161). Except for the least common combination MMF / Tacrolimus (4 patients), anemia was observed in the three other regimens (Fig. 3).

## Discussion

The main result was that no differences in PV and RCV were found between HTX and CON. For the first time, we present quantitative measurements of Hbmass and PV in the context of coincidental anemia in the long-term after HTX and showed that anemia occurs in a considerable number of patients as true anemia without a clear association with immunosuppression. As expected, based on the available studies in other groups of patients [26–28], the applicability of aCORM was safe in transplanted patients. The increase of [COHb] was comparable to existing data in a clinical setting [27, 28] and there were no unwanted or harmful effects.

Several methods are available for quantitative assessment of total BV, which is often considered laborious and generally not applied in routine diagnostics. In addition to the use of radioactive tracers in indicator-dilution techniques [9, 10], inhaled CO is a tracer to determine hemoglobin mass (Hbmass) and derivative RCV and PV. CO rebreathing was first used for determination of Hbmass more than 100 years ago [29] and was modified in different protocols of which two were predominantly applied in the recent decade as described by Burge and Skinner [16] and Schmidt and Prommer as the abbreviated form (aCORM) [17]. With Hbmass being the measured variable from which intravascular volumes are derived, it was shown that these volumes are closely correlated (r = 0.97) to those determined by radioactive labeling techniques [30]. As CO rebreathing allowed a detection of the withdrawal of small quantities of blood it can be considered as precise [17, 31] and less time consuming compared to radiolabeling techniques [26, 32]. Its application was safely possible in several groups of patients with anemia [26], coronary artery disease [27], chronic heart failure [11, 28], liver disease [33] as well as in the preoperative setting [34].

In comparison to the few existing studies in the field, we were able to examine three times as many heart transplant recipients and use an equally large matched control group. Although only of observational quality, another novelty was the inclusion of seven women in the final analysis, and we were able to study a greater variety of immunosuppressive regimens.

In HTX, PV was significantly increased in the small subset of women (+ 22% BSA-corrected PV) and there was a trend towards an expanded PV in the male subset (+ 6% BSA-corrected PV). Accordingly, the concentration-dependent variables venous [Hb] and Hct were significantly lower in HTX compared to CON but with presumably low clinical relevance judged upon the magnitude of the difference. Thus, our results support the previous findings of a PV expansion after transplantation also in so far as PV was lower in the subgroup analysis of patients treated with RAAS-inhibitors opposed to an increased PV in the non-RAAS-inhibition group. Although we present a cross-sectional approach, this is in line with previous work demonstrating a PV reduction of 12% upon administration of high-dose captopril [5]. Braith et al. concluded that fluid retention is partly engendered by a failure to reflexively suppress the RAAS when heart transplant recipients become hypervolemic [5].

In the past, immunosuppressant agents such as cyclosporine were also discussed to contribute to an expanded PV [5]. In contrast, despite receiving equivalent amounts of cyclosporine, Braith et al. presented six normovolemic liver transplant recipients compared to 11 hypervolemic heart transplant recipients [3]. In good correspondence with his report, we did not find an effect of the type of immunosuppressant regimen on PV.

The rate of diuretic therapy was 53% in the HTX group, which might have obscured PV expansion in some patients. However, if tested against HTX patients without diuretic therapy, there was no difference in PV, RCV or Hbmass. In a previous study of CHF patients, recompensation in response to diuretic therapy leads to a relatively small change in quantitative BV, most likely as a result of transcapillary shifting from the interstitial to the intravascular compartment [35].

As an analogy, applying aCORM in a group of CHF patients with reduced EF in our laboratory [11], an average PV (corrected for BSA) of 2069 ± 400 ml/m^2^ was found compared to 1919 ± 208 ml/m^2^ in this study demonstrating an about 8% lower PV in HTX compared to CHF. It could be hypothesized that over time, the pathophysiologic BV regulations known in CHF are no longer active after heart transplantation. Thus, based on our data, heterogeneity of PV distribution in heart transplant recipients becomes evident and PV expansion is perhaps less common than previously described. Interestingly, our HTX group yielded an elevated proBNP in line with previous observations [36] without evidence for volume overload.

### Anemia in heart transplant recipients

Particular attention should be given to anemia after heart transplantation due to its potential effect on long-term prognosis [12, 13]. The quantitative measurement of Hbmass as well as RCV and PV is important as it helps to distinguish true anemia from hemodilution. We opted to use WHO criteria [23] for the definition of anemia [13]. It seems there is a temporal dependence of the development of anemia with high prevalence postoperatively from 90% at discharge [37] to 78% six weeks after transplantation [38] and decrease in the further course when summarizing the few available studies. However, even 115 to 120 months after transplantation, anemia rates of 26% [37] to 41% [13] were reported. At a mean follow-up of 76 months after transplantation, we found a slightly lower anemia prevalence of 19%, which might have influenced the results.

For the first time, we are able to determine Hbmass and RCV in anemic heart transplant recipients. Opposed to pseudoanemia in CHF [7], true anemia is more prevalent after transplantation. Our anemic HTX patients had lower iron levels and transferrin saturation indicating possible mild iron deficiency although erythrocytes were normocytic and normochromic. Interestingly, this was not associated with renal function that was previously identified as a predisposing factor [13, 37]. As a limitation, our HTX subjects showed only moderate renal impairment with five HTX patients yielding a GFR < 40 ml/min. We further acknowledge that the analysis of anemia would have been more informative if performed earlier after HTX.

There are heterogeneous data concerning the immunosuppressant regimen as mycophenolate mofetil was found to be a predisposing factor for anemia [37] as well as sirolimus [39]. On the other hand, immunosuppressants (e.g. calcineurin inhibitors vs. mTOR-inhibitors) did not seem to affect the prevalence of anemia [13, 15]. To this end, evaluating four different immunosuppressant regimens, each used in a smaller number of subjects, we did not see an effect on the quantitative measures Hbmass and RCV.

In addition, as further non-anemic heart transplant recipients had similar BSA-corrected Hbmass and RCV compared to anemic subjects, it becomes obvious for the clinician that concentration-based parameters [Hb] and Hct do not adequately reflect intravascular volume status [26]. However, it is important to note that it remains difficult to define an individual normal Hbmass or RCV.

### Limitations

Interpretation of the impact of immunosuppression seems limited by the small numbers and the most common immunosuppression regimen in this patient cohort (everolimus/cyclosporine) which might be considered not reflective of the greater transplant community. Furthermore, the number of female and anemic subjects is low, which limits the significance of the analyses. Another possible limitation to the direct comparison with the few previous studies after heart transplantation [3, 4] is the introduction of a further method for quantitative BV measurement. As there is no consensus on what the standard method for predicting normal BV should be, we have used the control group of healthy subjects. In addition, various methods of adjustment of BV data for body size have been applied. Among others [7, 40], we applied BSA for adjustment as recommended by Pearson et al. [21] to present data for better comparison to the control group.

Finally, we studied compensated heart transplant recipients. It would have been interesting to focus on patients that re-developed heart failure or require hospitalization. This seems important, as data exists even in nonedematous CHF patients linking unrecognized hypervolemia to increased cardiac filling pressures and worse patient outcomes [41]. In the future, it would be of great interest of conducting a longitudinal study following heart transplant recipients with repetitive BV measurements over time, comparing those who develop heart failure to those who do not.

## Conclusions

There was no difference in blood volumes and Hbmass between HTX patients and control subjects in the long-term after heart transplantation. This is the first study applying CO rebreathing in a larger group of patients after heart transplantation. Based on our data, we hypothesize that the pathophysiologic BV regulation in CHF does not seem to be longer active in long-term HTX recipients. While PV was not significantly different from CON it may show a more heterogeneous distribution after HTX without a clear expansion. Anemia occurs in a considerable number of patients as true anemia that is suggestive of anemia in chronic disease and functional iron deficiency [15, 39]. Various immunosuppressant regimens did not affect the absolute measures Hbmass, RCV, or PV six years after heart transplantation in this group of stable heart transplant recipients.

## Data Availability

The datasets generated and/or analysed during the current study are not publicly available but are available from the corresponding author on reasonable request.

## Abbreviations

aCORM: abbreviated carbon monoxide rebreathing method
BSA: body-surface area
BV: blood volume
CHF: chronic heart failure
CO: carbon monoxide
[COHb]: carboxyhemoglobin concentration
CON: control group
CRP: c-reactive protein
EF: ejection fraction
[Hb]: hemoglobin concentration
Hbmass: total hemoglobin mass
Hct: hematocrit
HTX: heart transplant recipients
ICSH: International Council for Standardization in Hematology
MCHC: mean corpuscular hemoglobin concentration
MMF: mycophenolate mofetil
PV: plasma volume
RAAS: renin-angiotensin-aldosterone system
RCV: red cell volume
WHO: World Health Organization

## References

[1] Miller WL (2016). Fluid volume overload and congestion in heart failure: time to reconsider pathophysiology and how volume is assessed. Circ Heart Fail.

[2] Carry BJ, Katz SD (2018). Subclinical volume overload across the Spectrum of heart failure: lessons from Total blood volume measurements. J Card Fail.

[3] Braith RW, Mills RM, Wilcox CS, Convertino VA, Davis GL, Limacher MC (1996). Fluid homeostasis after heart transplantation: the role of cardiac denervation. J Heart Lung Transplant Off Publ Int Soc Heart Transplant.

[4] Bellet M, Cabrol C, Sassano P, Léger P, Corvol P, Ménard J (1985). Systemic hypertension after cardiac transplantation: effect of cyclosporine on the renin-angiotensin-aldosterone system. Am J Cardiol.

[5] Braith RW, Mills RM, Wilcox CS, Mitchell MJ, Hill JA, Wood CE (2000). High dose angiotensin-converting enzyme inhibition prevents fluid volume expansion in heart transplant recipients. J Am Coll Cardiol.

[6] Gunton RW, Paul W (1955). Blood volume in congestive heart failure. J Clin Invest.

[7] Adlbrecht C, Kommata S, Hülsmann M, Szekeres T, Bieglmayer C, Strunk G (2008). Chronic heart failure leads to an expanded plasma volume and pseudoanaemia, but does not lead to a reduction in the body’s red cell volume. Eur Heart J.

[8] Miller WL, Mullan BP (2016). Volume overload profiles in patients with preserved and reduced ejection fraction chronic heart failure. JACC Heart Fail.

[9] Miller WL, Albers DP, Gansen DN, Mullan BP (2018). Intravascular volume profiles in patients with class I and II systolic heart failure: heterogeneity and volume overload are common even in mild heart failure. J Card Fail.

[10] David M, Carsten L, Frank R, Flammer Andreas J (2017). True anemia―red blood cell volume deficit―in heart failure. Circ Heart Fail.

[11] Ahlgrim C, Birkner P, Seiler F, Wrobel N, Grundmann S, Bode C, Pottgiesser T (2020). Increased red cell volume is a relevant contributing factor to an expanded blood volume in compensated systolic chronic heart failure. J Card Fail.

[12] Müller HM, Aigner R, Horina JH, Rehak P, Lang T, Iberer F, Wasler A, Petutschnigg B, Allmayer T, Grasser B, Prenner G, Schaffellner S, Tscheliessnigg KH (1998). Mild chronic anemia following heart transplantation: a syndrome with prognostic relevance?. Transpl Int.

[13] Przybylowski P, Malyszko J, Malyszko J (2009). Anemia is a predictor of outcome in heart transplant recipients. Transplant Proc.

[14] Gleissner CA, Murat A, Schäfer S, Klingenberg R, Koch A, Remppis A (2004). Reduced hemoglobin after heart transplantation is no independent risk factor for survival but is associated closely with impaired renal function. Transplantation..

[15] Müller HM, Horina JH, Kniepeiss D, Tripolt MB, Stadelbauer V, Schweiger M, Tscheliessnigg KH (2001). Characteristics and clinical relevance of chronic anemia in adult heart transplant recipients. Clin Transpl.

[16] Burge CM, Skinner SL (1995). Determination of hemoglobin mass and blood volume with CO: evaluation and application of a method. J Appl Physiol Bethesda Md 1985.

[17] Schmidt W, Prommer N (2005). The optimised CO-rebreathing method: a new tool to determine total haemoglobin mass routinely. EurJApplPhysiol..

[18] Ahlgrim C, Schumacher YO, Wrobel N, Waller CF, Pottgiesser T (2014). Application of the optimized CO-rebreathing method for determination of hemoglobin mass in patients with polycythemia vera. Ann Hematol.

[19] Heinicke K, Wolfarth B, Winchenbach P, Biermann B, Schmid A, Huber G (2001). Blood volume and hemoglobin mass in elite athletes of different disciplines. IntJSports Med.

[20] Chaplin H, Mollison PL, Vetter H (1953). The body/venous hematocrit ratio: its constancy over a wide hematocrit range. JClinInvest..

[21] Pearson TC, Guthrie DL, Simpson J, Chinn S, Barosi G, Ferrant A (1995). Interpretation of measured red cell mass and plasma volume in adults: expert panel on radionuclides of the International Council for Standardization in Haematology. BrJHaematol..

[22] DuBois D, Dubois EF (1916). A formula to estimate the approximate surface area if height and weight be known. Arch Intern Med.

[23] WHO. Haemoglobin concentrations for the diagnosis of anaemia and assessment of severity. Vitamin and Mineral Nutrition Information System. Geneva, World Health Organization, 2011 (WHO/NMH/NHD/MNM/11.1) (http://www.who.int/vmnis/indicators/haemoglobin. pdf, accessed May 03 2020).

[24] Levey AS, Bosch JP, Lewis JB, Greene T, Rogers N, Roth D (1999). A more accurate method to estimate glomerular filtration rate from serum Creatinine: a new prediction equation. Ann Intern Med.

[25] Lang RM, Badano LP, Mor-Avi V, Afilalo J, Armstrong A, Ernande L, Flachskampf FA, Foster E, Goldstein SA, Kuznetsova T, Lancellotti P, Muraru D, Picard MH, Rietzschel ER, Rudski L, Spencer KT, Tsang W, Voigt JU (2015). Recommendations for cardiac chamber quantification by echocardiography in adults: an update from the American Society of Echocardiography and the European Association of Cardiovascular Imaging. Eur Heart J - Cardiovasc Imaging.

[26] Otto JM, Plumb JOM, Clissold E, Kumar S, Wakeham DJ, Schmidt W, et al. Hemoglobin concentration, total hemoglobin mass and plasma volume in patients: implications for anemia. Haematologica. 2017;102(9):169680. 10.3324/haematol.2017.169680 PMC5685237 28596281

[27] Karlsen T, Leinan IM, Aamot I, Hå D, StØylen A (2016). Safety of the co-rebreathing method in patients with coronary artery disease. Med Sci Sports Exerc.

[28] Ahlgrim C, Birkner P, Seiler F, Grundmann S, Baumstark MW, Bode C, Pottgiesser T (2018). Applying the optimized CO rebreathing method for measuring blood volumes and hemoglobin mass in heart failure patients. Front Physiol.

[29] Haldane J, Smith JL (1900). The mass and oxygen capacity of the blood in man. J Physiol.

[30] Thomsen JK, Fogh-Andersen N, Bülow K, Devantier A (1991). Blood and plasma volumes determined by carbon monoxide gas, 99mTc-labelled erythrocytes, 125I-albumin and the T 1824 technique. Scand J Clin Lab Invest.

[31] Pottgiesser T, Specker W, Umhau M, Dickhuth HH, Roecker K, Schumacher YO (2008). Recovery of hemoglobin mass after blood donation. Transfusion (Paris).

[32] Siebenmann C, Keiser S, Robach P, Lundby C (2017). CORP: the assessment of total hemoglobin mass by carbon monoxide rebreathing. J Appl Physiol.

[33] Plumb JOM, Otto JM, Kumar SB, Wright M, Schmidt W, Grocott MPW, Montgomery HE (2020). Application of the optimized carbon monoxide rebreathing method for the measurement of total haemoglobin mass in chronic liver disease. Physiol Rep.

[34] Otto JM, Plumb JOM, Wakeham D, Clissold E, Loughney L, Schmidt W, Montgomery HE, Grocott MPW, Richards T (2017). Total haemoglobin mass, but not haemoglobin concentration, is associated with preoperative cardiopulmonary exercise testing-derived oxygen-consumption variables. Br J Anaesth.

[35] Miller WL, Mullan BP (2014). Understanding the heterogeneity in volume overload and fluid distribution in decompensated heart failure is key to optimal volume management. JACC Heart Fail..

[36] Talha S, Marco PD, Doutreleau S, Rouyer O, Piquard F, Geny B (2008). Does circulating BNP normalize after heart transplantation in patients with normal hemodynamic and right and left heart functions?. Clin Transpl.

[37] Cursack GC, Crespo-Leiro MG, Paniagua-Martín MJ, Muñiz J, Naya C, Grille Z, Rodríguez JA, Marzoa R, Barge E, Ríos R, Estévez F, Cuenca JJ, Juffé-Stein A, Castro-Beiras A (2007). Chronic Anemia in heart transplant patients: prevalence, predisposing factors and prognostic significance. Rev Esp Cardiol Engl Ed.

[38] Taegtmeyer AB, Rogers P, Breen JB, Barton PJ, Banner NR, Yacoub MH (2008). The effects of pre- and post-transplant Anemia on 1-year survival after cardiac transplantation. J Heart Lung Transplant.

[39] McDonald MA, Gustafsson F, Almasood A, Barth D, Ross HJ (2010). Sirolimus is associated with impaired hematopoiesis in heart transplant patients? a retrospective analysis. Transplant Proc.

[40] Nijst P, Verbrugge FH, Bertrand PB, Martens P, Dupont M, Drieskens O, Penders J, Tang WHW, Mullens W (2017). Plasma volume is Normal but heterogeneously distributed, and true Anemia is highly prevalent in patients with stable heart failure. J Card Fail.

[41] Androne AS, Hryniewicz K, Hudaihed A, Mancini D, Lamanca J, Katz SD (2004). Relation of unrecognized hypervolemia in chronic heart failure to clinical status, hemodynamics, and patient outcomes. AmJCardiol..

